# Strain-induced quantum Hall phenomena of excitons in graphene

**DOI:** 10.1038/s41598-022-06486-z

**Published:** 2022-02-22

**Authors:** Oleg L. Berman, Roman Ya. Kezerashvili, Yurii E. Lozovik, Klaus G. Ziegler

**Affiliations:** 1 Physics Department, New York City College of Technology, The City University of New York, Brooklyn, NY 11201 USA; 2 The Graduate School and University Center, The City University of New York, New York, NY 10016 USA; 3 Institute of Spectroscopy, Russian Academy of Sciences, Troitsk Moscow, Russia 142190; 4 Research University Higher School of Economics, Moscow, Russia 101000; 5 Institut für Physik, Universität Augsburg, 86135 Augsburg, Germany

**Keywords:** Nanoscience and technology, Physics

## Abstract

We study direct and indirect pseudomagnetoexcitons, formed by an electron and a hole in the layers of gapped graphene under strain-induced gauge pseudomagnetic field. Since the strain-induced pseudomagnetic field acts on electrons and holes the same way, it occurs that the properties of single pseudomagnetoexcitons, their collective effects and phase diagram are cardinally different from those of magnetoexcitons in a real magnetic field. We have derived wave functions and energy spectrum of direct in a monolayer and indirect pseudomagnetoexcitons in a double layer of gapped graphene. The quantum Hall effect for direct and indirect excitons was predicted in the monolayers and double layers of gapped graphene under strain-induced gauge pseudomagnetic field, correspondingly.

## Introduction

In this study we focus on the influence of strain on the properties of excitons and their collective properties in mono and double layers of gapped graphene. It was predicted that an in-plane distortion of the graphene lattice due to non-uniform strain is equivalent to creation of large, nearly uniform pseudomagnetic fields (PMF), acting on electrons, which lead to the formation of Landau levels (LLs) and zero magnetic field Quantum Hall effect (QHE) for electrons^1–5^. Note that the Quantum Hall phenomena in graphene and graphene based structures in a high magnetic field attracted the great interest^6–8^. Landau quantization of the electronic spectrum for highly strained nanobubbles was experimentally observed, and PMFs in excess of $300\ \mathrm {T}$ have been measured^9^. A 2D electron gas (2DEG) in graphene in a high strain-induced PMF attracted strong interest very recently^10^. Topological features of neutral particle-hole pair excitations of correlated quantum anomalous Hall (QAH) insulators have been studied with application to broken-symmetry spontaneous QAH insulators in substrate aligned magic-angle twisted bilayer graphene^11^. The optical properties of interlayer excitons have been analyzed in heterobilayer transition metal dichalcogenides, where the moire pattern is introduced in heterostructures, in comparison with that introduced by twisting (and/or lattice mismatch)^12^.

We consider excitons in the QHE regime, where the uniform pseudomagnetic field can be produced by strain^13^. The gap and the strain field have a different origin in graphene: The gap appears when we break the sublattice symmetry (e.g., by doping with hydrogen), whereas the strain is caused by smooth deformations of the graphene sheet. Therefore, both effects can be engineered independently.

Consider strained gapless graphene. Due to the symmetry of its lattice, graphene has two independent chiral valleys: *K* and $K^{^{\prime }}$. Clockwise and counterclockwise polarized photons are absorbed in different valleys. Strain-induced effective pseudomagnetic field has the opposite direction in the *K* and $K^{^{\prime }}$ valleys^3^. This is due to the fact that there is no violation of invariance with respect to time reversal symmetry in the strained graphene, in contrast to the case with a real magnetic field.

When photons from beam with circular polarization are absorbed, two types of pairs of electrons and holes arise in one of the valleys: an electron with spin up and a hole with spin down, or vice versa. As a result of the relaxation of electrons and holes with the emission of phonons, two types of intravalley excitons, that are bound state of the electron and hole from the same valley, arise: singlet and triplet state excitons. Due to the spin conservation during recombination, triplet excitons are long-lived, dark, and singlet excitons are bright. Consider now pumping by a linearly polarized light beam. A linearly polarized photon is a quantum superposition of two opposite circular polarizations. Therefore, as a result of the absorption of photons from a light beam with linear polarization, pairs of electrons and holes appear in both valleys. After relaxation with emission of phonons, intravalley singlet and triplet excitons appear the same valley *K* or $K^{^{\prime }}$, namely, two singlet ones, with a spin up electron and a spin down hole and vice versa, as well as two triplet, with a spin up electron and a spin up hole and with a spin down electron and a spin down hole. Therefore, there are eight types of intravalley excitons. It is worth noting that the recombination of intervalley exciitons with photons radiation is forbidden due to a large momentum between the valleys in comparison with momentum of a photon, i.e. intervalley exciitons are dark exciitons. By taking into account spins, there are also eight types intervalley excitons with an electron from the *K* valley and a hole from the $K^{^{\prime }}$ valley or vice versa. Due to the symmetry of the *K* and $K^{^{\prime }}$ valleys and the negligible spin-orbit interaction in graphene, all sixteen types of excitons have the same energy. The latter means that the degeneracy factor is 16. In particular, for this reason, intravalley excitons have the same binding energy and the same dependence of the energy on momentum. In a real magnetic field, singlet excitons in the *K* and $K^{^{\prime }}$ valleys also have the same binding energy, the same continuous dependence on the magnetic momentum, and the conserved value in the magnetic field. The conservation is associated with the invariance of the system with respect to translation and gauge invariance^17^. As a result, the 2D magnetoexciton spectrum is a set of allowed energy bands separated by distances between Landau levels^14^. Due to this, singlet intravalley excitons have the same collective properties, and they form a Bose-Einstein condensate (BEC) at low temperatures (ideal for spatially direct excitons in a single-layer system, see^15,16^ and references therein). For triplet intravalley and intervalley excitons, one should take into account the interaction of the spin with the real magnetic field, which leads to the Zeeman splitting.

It is important to note that in intravalley excitons electrons and holes are affected by the same PMF inherent in a given valley. In intervalley excitons, opposite PMFs acts on electrons and holes. This leads to completely different properties of intravalley and intervalley excitons.

Below we show that since the strain induced PMF, in contrast to a real magnetic field, acts on the different electric charges from two different valleys in the same way, it leads to novel effects for excitons in PMFs as considered below. These effects are: i. a discrete energy spectrum of excitons in a PMF in contrast to excitons in a real magnetic field, characterized by the continuous energy dependence on the magnetic momentum on each LL^14,17^; ii. rich Quantum Hall phenomena for excitons in a PMF which are absent for excitons in a real magnetic field.

In this work we calculate the LLs of direct and indirect excitons in mono and double layers of gapped strained graphene, respectively, in the presence of a PMF. Such excitons we refer to as PMEs. We consider the existence of the Integer and Fractional Quantum Hall effects and the state of composite fermions at $\nu = 1/2$ for the PMEs. We propose also the experimental methods to observe these phenomena.

This article is organized in the following way. In "Strain-induced pseudomagnetoexcitons" section we consider the formation of PMEs in strained graphene. The Landau levels for the non-interacting electron and hole in strain-induced PMF and the wave functions and energy leveles of a PME are presented in “Non-interacting electrons and holes in a strain-induced PMF” and “The energy of a PME” sections, respectively. In “Strain-induced quantum hall phenomena for pmes” in section we discuss strain-induced Quantum Hall phenomena for PMEs. Discussions and conclusions related to collective properties of the PMEs follow in “Discussion and conclusions” section.

## Strain-induced pseudomagnetoexcitons

A strain-induced gauge field can cause a large PMF to appear for certain selected geometries of the applied strain in graphene monolayers^3^. Specifically, a modest strain field with triangular symmetry results in the motion of the charge carriers similar to their motion in the approximately uniform, quantizing magnetic field up of tens of Tesla^1^.

In this section we provide the theoretical formalism for the description of Mott-Wannier excitons in a monolayer or in a double layer of gapped strained graphene. We consider for both cases an equal number of electrons and holes, where in the case of the double layer the electrons are in one layer, the holes are in the other layer. Then we add strain, which induces a PMF $B$ perpendicular to the layers^3^. We further assume that we apply a strain field that causes a uniform PMF^1^.

In the following we will study the two-body problem of an electron-hole pair in gapped graphene mono and double layers, subject to a strain-induced PMF.

Starting from the effective Dirac Hamiltonian for a single valley of gapped graphene in the presence of a strain field with components $A_{x}$ and $A_{y}$^3^, we consider the Dirac equation for a pair of an electron at position $\mathbf {r}_{e}$ and a hole at position $\mathbf {r}_{h}$ attracting via $V\left( \left| \mathbf {r}_{e}-\mathbf {r}_{h}\right| \right)$ potential. The corresponding Dirac equation for the spinor $\Psi$ reads

1 $$\begin{aligned} \left( H_{0}+\ V\left( \left| \mathbf {r}_{e}-\mathbf {r}_{h}\right| \right) \right) \Psi =\mathcal {E}\Psi ,\ \ \Psi =\left( \begin{array}{c} \psi _{1}(\mathbf {r}_{e},\mathbf {r}_{h}) \\ \psi _{2}(\mathbf {r}_{e},\mathbf {r}_{h}) \end{array} \right) , \end{aligned}$$

where the Dirac Hamiltonian of the non-interacting electrons and holes is

2 $$\begin{aligned} H_{0}=v_{F}\sum _{j=e,h}\left( \begin{array}{cc} 2 \Delta _{j}/v_{F} &{} i\hbar \partial _{x_{j}}+A_{x}(\mathbf {r}_{j})+\hbar \partial _{y_{j}}-iA_{y}(\mathbf {r}_{j}) \\ i\hbar \partial _{x_{j}}+A_{x}(\mathbf {r}_{j})-\hbar \partial _{y_{j}}+iA_{y}(\mathbf {r}_{j}) &{} -2 \Delta _{j}/v_{F} \end{array} \right) . \end{aligned}$$

In Eq. (2) $v_{F}$ is the Fermi velocity, while $\Delta$ is the gap between the conductance and valence bands in the single-particle spectrum.

Next we consider the situation, when the energies of the electrons in the conduction band above the gap and the energy of the holes in the valence bands below the gap are significantly smaller than the energy gap between the bands. Below we are using the gaps that are satisfying the condition that the gap is larger than the separation between LLs. The quadratic dispersion in the absence of strain induced pseudomagnetic field is valid for wavevectors *k* up to $k^{\prime }$ ($k^{\prime }$ is is the maximally admitted wavevector where at least several LL are present) when $\hbar v_{F}k^{\prime }$ is smaller than the gap. Then we consider pseudomagnetic fields, for which at least two LLs exists in this energy regime. By decreasing the strain field, we can create more LLs in this regime. Then we can neglect small terms in the Dirac Eq. (1) with the Hamiltonian $H_{0}$ and follow the standard approach (see, e.g., Ref.^18^) obtain the Schrödinger equation.

Now we introduce the effective Hamiltonian operators of an electron and a hole taken either from the same valley or from different valleys in the strain-induced PMF. The Hamiltonian operator of electrons near the bottom of the conduction band can be written as

3 $$\begin{aligned} \hat{\mathcal {H}}_{c} = \frac{1}{2m_{c}} \sum _{\mathbf {p}} \left( \mathbf {p} - \mathbf {A}_{c} \right) ^{2}c_{\mathbf {p}}^\dagger c_{\mathbf {p}} , \end{aligned}$$

and near the top of the valence band as

4 $$\begin{aligned} \hat{\mathcal {H}}_{v} = \frac{1}{2m_{v}} \sum _{\mathbf {p}} \left( \mathbf {p} - \mathbf {A}_{v} \right) ^{2} c_{\mathbf {p}}^\dagger c_{\mathbf {p}} , \end{aligned}$$

where $\mathbf {p}$ is the momentum of an electron, $c_{\mathbf {p}}^\dagger$ and $c_{\mathbf {p}}$ are electron creation and annihilation operators; $m_{c(v)}$ ($m_{c} > 0$; $m_{v} < 0$) and $\mathbf {A}_{c(v)}$ are the effective masses and strain-induced vector potentials for electrons near the bottom of the conduction band and near the top of the valence band, correspondingly.

The particle-hole transformation of $c\rightarrow h^{\dagger }$ near the top of the valence band leads to the Hamiltonian for holes

$$\begin{aligned} \hat{\mathcal {H}}_{h}=\frac{1}{2m_{h}}\sum _{\mathbf {p}}\left( \mathbf {p}+ \mathbf {A}_{h}\right) ^{2}h_{\mathbf {p}}^{\dagger }h_{\mathbf {p}} , \end{aligned}$$

where $m_{h}=-m_{v}$ is the mass of a hole, $h_{\mathbf {p}}^{\dagger }$ and $h_{\mathbf {p}}$ are hole creation and annihilation operators, and $\mathbf {A} _{h}$ is the strain-induced vector potential, acting on a hole. If an electron and a hole are in the same valley, then we get $\mathbf {A}_{h}= \mathbf {A}_{c}$, while for an electron and a hole taken from different valleys, one has $\mathbf {A}_{h}=-\mathbf {A}_{c}$, since contrary to a real magnetic field, the strain-induced PMF does not break the time-reversal symmetry^3^. Therefore, one has for the Hamiltonian operator of the holes, taken from the same valley as the electrons,

$$\begin{aligned} \hat{\mathcal {H}}_{h}=\frac{1}{2m_{h}}\sum _{\mathbf {p}}\left( \mathbf {p}+ \mathbf {A}_{c}\right) ^{2}h_{\mathbf {p}}^{\dagger }h_{\mathbf {p}} , \end{aligned}$$

while the Hamiltonian operator of the holes taken from a different valley than that of the electrons, is written as

$$\begin{aligned} \hat{\mathcal {H}}_{h}=\frac{1}{2m_{h}}\sum _{\mathbf {p}}\left( \mathbf {p}- \mathbf {A}_{c}\right) ^{2}h_{\mathbf {p}}^{\dagger }h_{\mathbf {p}}\ . \end{aligned}$$

From these considerations we conclude that the PMF acts differently, depending on whether the electron and the hole are from the same or from different valleys. In other words, the sign of the vector potential changes when we change one of the valleys, either for the electrons or for the holes. Thus, the PMFs acts on an electron and a hole from the same valley in opposite direction like a real magnetic field, while it acts on an electron and a hole from the different valleys in the same direction, in contrast to a real magnetic field.

**Figure 1:**
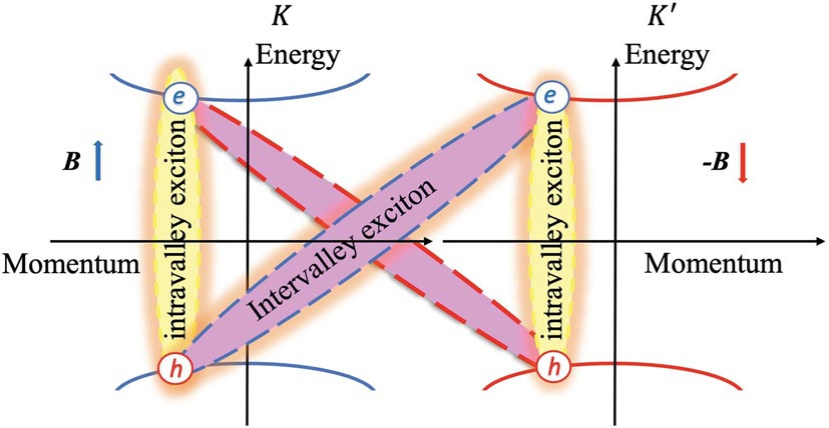
The schematic band structure and electronic dispersions in the graphene monolayer for bright intravalley and dark intervalley pseudo magnetoexcitons in the *K* and $K^{^{\prime }}$ valleys. The second intervalley PME is shown by the dashed curve.

To make our statements more clearly, we have to look at the single-particle energy spectrum shown in Fig. 1. As can be seen, there can be two types of excitons: i. the intravalley exciton formed by an electron and a hole in the same *K* (or $K^{^{\prime }}$) valley, and ii. the intervalley exciton formed by an electron in the *K* (or $K^{^{\prime }}$) valley and a hole in the $K^{^{\prime }}$ (or *K*) valley. From the different electron-hole symmetries exhibited in the single-particle energy spectra, we may expect that the intravalley and intervalley exciton states should display different magnetic-field dependencies.

The processes of absorption and emission of photons in graphene (during the recombination of electrons and holes or excitons) are governed by the following conservation laws: the laws of conservation of energy and momentum and the law of conservation of spins (due to the negligible spin-orbit interaction in graphene). The laws of conservation of energy and momentum lead to the fact that when a photon with circular polarization is absorbed, an electron appears in the upper band and a hole in the lower band only in the same valley, since the photon momentum is much less than the difference in momentum between the centers of the valleys. For the same reason, an intervalley exciton, the bound state of an electron and a hole from different valleys cannot recombine with the emission of a photon. The time scales for intravalley and intervalley excitons are very different. The time of life for intervalley excitons is several orders of magnitude longer than for intravalley excitons in the absence of energy and momentum conservation. Therefore, the probability of recombination with phonon participation is too small. Thus, after short time only intervalley excitons can be observed.

Let us now consider peculiarities of excitons in graphene in a pseudomagnetic field. First, the PMF induced by strain does not interact with the spin. Thus, the singlet and triplet intravalley excitons have the same energy. This take place also for the singlet and triplet intervalley excitons. In PMF intravalley excitons have the same properties as in a real magnetic field. This is due to the fact that the quantity conserved in a pseudomagnetic field (pseudomagnetic momentum) has the same form as in a real magnetic field. As a result, PMEs have the same energy dependence on pseudomagnetic momentum as magnetoexcitons on magnetic momentum. Their spectrum is also a set of bands with allowed energy, separated by distances between Landau levels in a pseudomagnetic field. As a result, intravalley PMEs have the same collective properties as magnetoexcitons, that is, they form a BEC at low temperatures. Based on this description, we conclude that the photoluminescence signal of the electron-hole recombination at very short time scale is due to the intravalley excitons. They form an intravalley exciton BEC, similar to the BEC of magnetoexcitons in a magnetic field^14–16^. This is due to the pseudomagnetic field that acts on an electron and a hole, forming an intravalley exciton, the same way. These PMEs can form a BEC.

A completely different situation takes place for intervalley pseudomagnetoexcitons. Due to the fact that in different valleys the PMF has a different direction, the pseudomagnetic momentum is fundamentally different from the magnetic momentum, namely, the pseudomagnetic field acts on electrons and holes in the same way. As a result, the spectrum of the PME becomes similar to the spectrum of a 2D electron in a real magnetic field: the spectrum is completely discrete. This leads to completely new collective properties of intervalley pseudomagnetic excitons. Namely, this leads to the manifestation of a number of phenomena, such as the Integer and Fractional Quantum Hall Effect and so on. Thus, in this Paper we will consider only excitons formed by an electron and a hole from different valleys.

As it is follows from above discussion, in stark contrast to the vector potential of the electromagnetic field, the strain induced effective vector potentials $\mathbf {A}(\mathbf {r}_{e})$ and $\mathbf {A}(\mathbf {r}_{h})$, acting on an electron and a hole from the different valleys, forming a PME, are not coupled to the charges of the particles and have the same sign in the Hamiltonian (2), and these potentials act on *e* and *h* the same way.

Let us mention that one can consider *e* and *h* in different layers, which form indirect^17^ PMEs in a graphene double layer. In this case, the application of different doping to the two graphene monolayers can lead to the formation of two different gaps in these graphene monolayers. In such a system the effective masses of electron and hole $m_{e(h)}=\Delta _{e(h)}/v_{F}^{2}$^19^, are not equal: $m_{e}\ne m_{h}$.

The Hamiltonian $\hat{\mathcal {H}}$ of an electron-hole pair, forming a PME in the strain-induced PMF, can be written as

5 $$\begin{aligned} \hat{\mathcal {H}} = \hat{\mathcal {H}}_{0} + V\left( \left| \mathbf {r}_{e} - \mathbf {r}_{h} \right| \right) , \end{aligned}$$

where the Hamiltonian $\hat{\mathcal {H}}_{0}$ of non-interacting an electron and a hole in the strain-induced PMF is given by

6 $$\begin{aligned} \hat{\mathcal {H}}_{0}=\frac{1}{2m_{e}}\left( -i\hbar \nabla _{\mathbf {r} _{e}}-\mathbf {A}(\mathbf {r}_{e})\right) ^{2}+\frac{1}{2m_{h}}\left( -i\hbar \nabla _{\mathbf {r}_{h}}-\mathbf {A}(\mathbf {r}_{h})\right) ^{2}, \end{aligned}$$

where $\mathbf {A}(\mathbf {r}_{1(2)})=\mathbf {B}\times \mathbf {r}_{1(2)}/2$ is the strain-induced effective vector potential, in cylindrical gauge, and $\mathbf {B}$ is the strain-induced PMF. Recently it has been shown that strain pseudomagnetic field in two twisted graphene or TMDC layers giving a moire pattern will couple to the valley magnetic moment and contributes a pseudo-Zeeman term^20,21^. We are not considering two twisted layers in the present work.

The energy of the $e-h$ attraction $V\left( \left| \mathbf {r}_{e}- \mathbf {r}_{h}\right| \right)$ in Eq. (8) can be described by the Rytova-Keldysh (RK) potential ^22,23^

7 $$\begin{aligned} V_{RK}(r)=-\frac{\pi ke^{2}}{2\kappa \rho _{0}}\left[ H_{0}\left( \frac{r}{ \rho _{0}}\right) -Y_{0}\left( \frac{r}{\rho _{0}}\right) \right] , \end{aligned}$$

or by the Coulomb interaction. In Eq. (7) $\kappa =(\epsilon _{1}+\epsilon _{2})/2$ describes the surrounding dielectric environment, $\epsilon _{1}$ and $\epsilon _{2}$ are the dielectric constants either below and above the monolayer, $H_{0}$ and $Y_{0}$ are the Struve and Bessel functions of the second kind, respectively, and $\rho _{0}$ is the screening length. The results of our calculations with the RK potential are compared with those of the Coulomb potential.

The Schrödinger equations for a ME in a magnetic field and for the PME in a PMF are invariant with respect to the magnetic translation and gauge transformations. This invariance for a magnetic field results in the conservation of the operator of the magnetic momentum of the ME $\hat{ \widetilde{\mathbf {P}}}=-i\hbar \nabla _{\mathbf {r}_{e}}-i\hbar \nabla _{ \mathbf {r}_{h}}-\frac{e\mathbf {B}_{0}\times \left( \mathbf {r}_{e}-\mathbf {r} _{h}\right) }{2}$, where $\mathbf {B}_{0}$ is the magnetic field, which commutes with the Hamiltonian and has the same eigenfunctions as the Hamiltonian^14,17,24^. The dependence of $\hat{\widetilde{ \mathbf {P}}}$ on the coordinate of the relative motion of an electron and a hole $\mathbf {r}=\mathbf {r}_{e}-\mathbf {r}_{h}$ leads to the continuous dependence of the ME energy and wavefunction on $\hat{\widetilde{\mathbf {P}}}$. Contrary to a magnetic field, in a PMF the simultaneous translation and gauge transformations leads to the operator of pseudomagnetic momentum $\hat{ \mathbf {P}}$:

8 $$\begin{aligned} \hat{\mathcal {H}} = \hat{\mathcal {H}}_{0} + V\left( \left| \mathbf {r}_{e} - \mathbf {r}_{h} \right| \right) . \end{aligned}$$

The difference between the expressions for $\hat{\widetilde{\mathbf {P}}}$ and $\hat{\mathbf {P}}$ is related to the fact that the PMF acts on *e* and *h* the same way due to the same sign for the pseudovector potential in Hamiltonian (6). Following the standard procedure for the separation of the center-of-mass and relative motion we introduce the coordinates of the center-of-mass $\mathbf {R}=\frac{m_{e}\mathbf {r}_{1}+m_{h}\mathbf {r}_{2} }{m_{1}+m_{2}}$ and relative motion $\mathbf {r}=\mathbf {r}_{1}-\mathbf {r}_{2}$. In these coordinates for the magnetic momentum operator of the magnetoexciton we have

9 $$\begin{aligned} \hat{\widetilde{\mathbf {P}}} = -i \hbar \nabla _{\mathbf {R}} - \frac{ e \mathbf {B}_{0} \times \mathbf {r}}{2}. \end{aligned}$$

Using these coordinates one can rewrite the operator of pseudomagnetic momentum $\hat{\mathbf {P}}$ as

10 $$\begin{aligned} \hat{\mathbf {P}} = -i \hbar \nabla _{\mathbf {R}} - \mathbf {B} \times \mathbf {R } - \frac{ \gamma \mathbf {B} \times \mathbf {r}}{2} , \end{aligned}$$

where

11 $$\begin{aligned} \gamma = \frac{m_{h} - m_{e}}{m_{h} + m_{e}} . \end{aligned}$$

Since in the case of equal electron and hole effective masses at $m_{e}=m_{h}$ one has $\gamma =0$, the third term in the r.h.s. of Eq. (10) vanishes, and the PMF acts only on the center-of-mass of an electron and a hole and does not affect on their relative motion.

Using the coordinates for the center-of-mass $\mathbf {R}$ and relative motion $\mathbf {r}$ of the electron-hole system and performing the detailed lengthly calculations given in Supplementary Material (SM) V A, the Hamiltonian (6) for non-interacting electron and hole can be written in terms of the operator $\hat{\mathbf {P}}$ as

12 $$\begin{aligned} \hat{\mathcal {H}}_{0} = \frac{1}{2M} \left[ \hat{\mathbf {P}}^{2} - \frac{1}{ m_{e}m_{h}}\left( \hbar M \nabla _{\mathbf {r}} - i \mathbf {S} (\mathbf {R}, \mathbf {r}) \right) ^{2}\right] , \end{aligned}$$

where

13 $$\begin{aligned} \mathbf {S}(\mathbf {R},\mathbf {r}) = \frac{\mathbf {B}}{2}\times \left[ (m_{h}-m_{e})\mathbf {R} + \frac{\left( m_{e}^{2} + m_{h}^{2}\right) \mathbf {r} }{M} \right] , \end{aligned}$$

where $M = m_{e} + m_{h}$ is the total exciton mass and the operator $\hat{ \mathbf {P}}^{2}$ is given by

14 $$\begin{aligned} \hat{\mathbf {P}}^{2} = \left[ -i \hbar \nabla _{\mathbf {R}} - \mathbf {B} \times \mathbf {R} - \frac{ \gamma \mathbf {B} \times \mathbf {r}}{2} \right] ^{2}. \end{aligned}$$

The operator $\hat{\mathbf {P}}^{2}$ commutes with the Hamiltonian for the non-interacting electro-hole pair in the PMF and shares the same eigenfunctions with the Hamiltonian (12). However, the eigenfunctions of $\hat{\widetilde{\mathbf {P}}}$ (10) and $\hat{ \mathbf {P}}$ (9) are different. We will see below that this implies a discrete spectrum for the PMEs contrary to a continuous spectrum for the magnetoexcitons. This implies that, in contrast to the 2D many-magnetoexciton system, the collective properties of a 2D many-PME system occur to be similar to a 2D electron system in a high magnetic field.

### Non-interacting electrons and holes in a strain-induced PMF

Now we study the eigenfunctions and eigenvalues of the Hamiltonian $\mathcal { H}_{0}$ (6). Following the detailed lengthly calculations given in SM V A, the eigenvalues of $\mathcal {H}_{0}$ of the non-interacting electron-hole pair in the strain-induced PMF can be presented as

15 $$\begin{aligned} \mathcal {E}_{0n,\tilde{n}} = E_{n} + \tilde{E}_{\tilde{n}} = \left( n + \frac{ 1}{2}\right) \hbar \omega _{c} + \left( \tilde{n} + \frac{1}{2}\right) \hbar \tilde{\omega }_{c}, \end{aligned}$$

where $\omega _{c} = 2 B/M$ and $\tilde{\omega }_{c} = \left( m_{e}^{2} + m_{h}^{2}\right) \mathbf {B}/M^{2}\mu$ are the cyclotron frequencies for the center-of-mass motion and the relative motion of the non-interacting electron and hole, respectively, while $n=0,1,2,\ldots$ and $\tilde{n} =0,1,2,\ldots$ are the quantum numbers. Moreover, according to the calculation in SM V A, for the eigenfunctions we get

16 $$\begin{aligned} \Psi _{n,m,\tilde{n},\tilde{m}}(\mathbf {R},\mathbf {r}) = \psi _{n,m}^{(0)}( \mathbf {R})\tilde{\varphi }_{\tilde{n},\tilde{m}}^{(0)}(\mathbf {r}) e^{i \gamma \left( \mathbf {B} \times \mathbf {r}\right) \cdot \mathbf {R}/2\hbar }, \end{aligned}$$

where $\gamma$ is defined by Eq. (11). The $\mathbf {R}$–dependent function $\psi _{n,m}^{(0)}(\mathbf {R})$ is the wavefunction for a free particle in the effective PMF 2**B** in the cylindrical gauge in Refs.^14,17,25^. The $\mathbf {r}$–dependent function $\tilde{\varphi }_{\tilde{n},\tilde{m}}^{(0)}(\mathbf {r})$ is the wavefunction for a free particle the effective PMF ${ {\tilde{\mathbf{B}}}} = \left( m_{e}^{2} + m_{h}^{2}\right) \mathbf {B}/M^{2}$ in the cylindrical gauge and it is

17 $$\begin{aligned} \tilde{\varphi }_{\tilde{n},\tilde{m}}^{(0)}(\mathbf {r}) = \left[ \frac{\tilde{ n}!}{2 \pi \left( \tilde{n}+ |\tilde{m}|\right) !}\right] ^{1/2} \frac{ \exp \left( i\tilde{m}\phi \right) }{l}\left( \frac{r}{\sqrt{2}l}\right) ^{| \tilde{m}|} L_{\tilde{n}}^{|\tilde{m}|}\left( \frac{r^{2}}{2l^{2}}\right) \exp \left( - \frac{r^{2}}{4l^{2}}\right) , \end{aligned}$$

where $l = \sqrt{\hbar /{\tilde{B}}}$ is the pseudomagnetic length and $L_{ \tilde{n}}^{|\tilde{m}|}$ are Laguerre polynomials.

In the case when $m_{e}=m_{h}\equiv m_{0}$ we have $\omega _{c}=\tilde{\omega } _{c}=B/m_{0}$, where $m_{0}=\Delta /v_{F}^{2}$ for the *e* and *h* masses. In this case from Eq. (15) we obtain a simple elegant expression for the energy of the non-interacting electron-hole pair in the strain-induced PMF:

18 $$\begin{aligned} \mathcal {E}_{0n,\tilde{n}} = E_{n} + \tilde{E}_{\tilde{n}} = \left( n + \tilde{n}+1\right) \hbar \omega _{c}, \end{aligned}$$

where $\omega _{c} = B/m_{0}$ is the cyclotron frequency for the motion of the center-of-mass and the relative motion of the non-interaction electron–hole pair, $n=0,1,2,\ldots$ and $\tilde{n} = 0,1,2,\ldots$ are the quantum numbers for the motion of the center-of-mass and the relative motion of a non-interacting electron –hole pair, respectively. Therefore, Eqs. (15) and (18) present the quantized eigenenergy of the non-interacting electron and hole in the strain induced PMF with non-equal and equal electron and hole masses, respectively. Thus, the energy levels of a non-interacting two-dimensional electron-hole system in a PMF are quantized into a discrete set of LLs with degeneracy proportional to the area of the system ^26^.

We use the expressions (15) and (18) to find the spectrum of the corresponding Schrödinger equation for the non-interacting electron-hole pair. Let us mention that for the non-interacting electron-hole pair in a high strain-induced PMF we obtain that the energy spectrum of both the motion of the center-of-mass and the relative motion are quantized.

In the case of the double layer when it is also possible that $m_{e}\ne m_{h}$, the energy of the non-interacting electron-hole pair in the strain-induced PMF can be found by using Eq. (15). In this case due to different band gaps in the first and the second graphene layer we have $m_{e}\ne m_{h}$. This leads to different cyclotron frequencies $\omega _{c}$ and $\tilde{\omega }_{c}$ for the motion of the center-of-mass and the relative motion of the non-interacting electron and hole.

If one acts by the Hamiltonian of the magnetoexciton on the eigenfunction of $\hat{\widetilde{\mathbf {P}}}$ and employs the certain variable change, the dependence of the resulting Hamiltonian on the eigenvalue $\widetilde{ \mathbf {P}}$ appears only in one term in this Hamiltonian. This term is responsible for the electron-hole Coulomb attraction as the replacement of $\mathbf {r}$ by $\mathbf {r} + \mathbf {r}_{0}$, where the continuously changing parameter $\mathbf {r}_{0}$ is directly proportional to the eigenvalue $\widetilde{\mathbf {P}}$, which can vary continuously from 0 to infinity^14,17^.

Therefore, while the energy spectrum of a magnetoexciton is discrete in the zeroth order with respect to the electron-hole attraction, the energy spectrum of a magnetoexciton becomes a continuous function of the eigenvalue $\widetilde{\mathbf {P}}$ in the first order perturbation theory with respect to the electron-hole Coulomb attraction^14,17^. The simultaneous invariance of the Schrödinger equation for a PME in the strain-induced PMF $\mathbf {B}$ with respect to the translation and the gauge transformations results in the conservation of $\hat{\mathbf {P}}^{2}$. The difference between the third terms of $\hat{\widetilde{\mathbf {P}}}$ and $\hat{\mathbf {P}}$ is caused by the fact that while the action of the magnetic field on particles depends on the value and sign of charge of a particle, the action of the strain-induced PMF on particles does not depend on the value and sign of charge of a particle. Therefore, the strain-induced PMF acts on an electron and a hole the same way contrary to the magnetic field, which acts on an electron and a hole differently. Thus, the eigenfunctions are different for magnetoexcitons and PMEs because the PMF acts the same way on *e* and *h* and the strain-induced PMF acts on a PME similar to the action of the magnetic field on two identical charged particles.

### The energy of a PME

Now let us find the energies of a PME in a mono and double layer of strained and gapped graphene in the presence of the $e-h$ attraction. Assuming that contribution of $V\left( \left| \mathbf {r}_{e}-\mathbf {r}_{2}\right| \right)$ to the energy of the PME (the binding energy of the PME) is small compared with the difference between the eigenvalues of $\hat{\mathcal {H}}$, we start with the zeroth order of the perturbation theory with respect to $V\left( \left| \mathbf {r}_{e}-\mathbf {r}_{h}\right| \right)$ and find the eigenvalues for (8).

**Figure 2:**
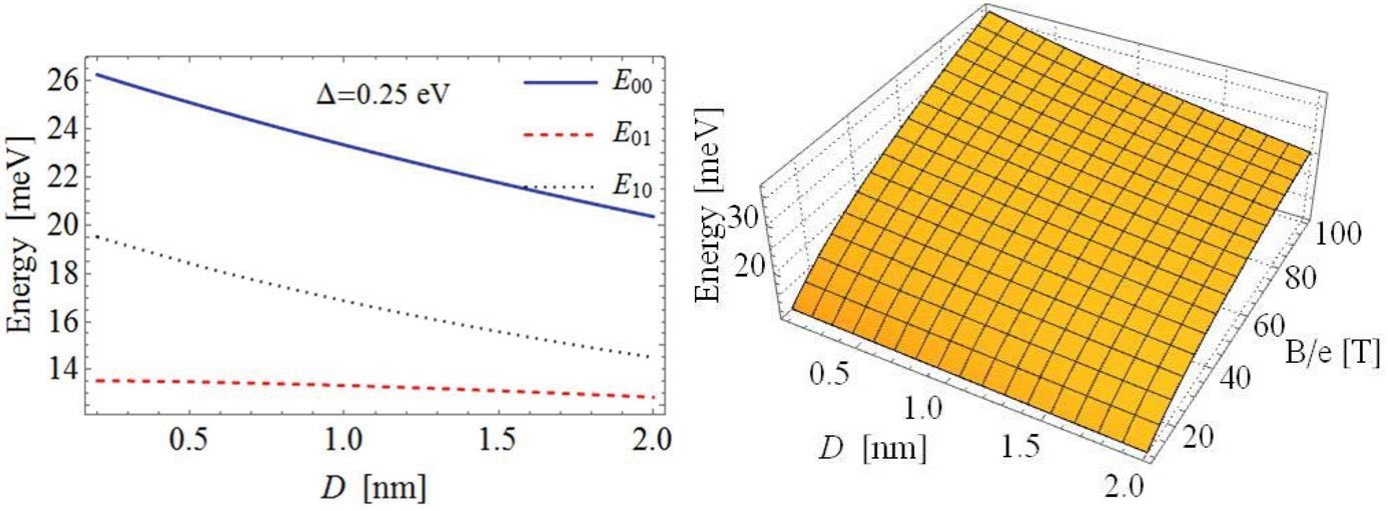
Left panel: the dependence of energies $E_{n,\tilde{n }}^{\prime }$ of indirect PMEs on the separation *D* between gapped graphene layers. Calculations performed for the value of magnetic length *l* that corresponds to $B/e=50\ \mathrm {T}$. Right panel: the dependence of the energies of indirect PMEs $E_{\tilde{n},\tilde{m}}^{\prime }$ on the separation *D* between gapped graphene layers and PMF *B*/*e*.

The attractive $e-h$ interaction we treat in the framework of the perturbation theory. Neglecting the transitions between different LLs, the first order perturbation with respect to $e-h$ attraction results in the following expression for the energy $E_{\tilde{n},\tilde{m}}^{\prime }$ of a PME:

19 $$\begin{aligned} E_{\tilde{n},\tilde{m}}^{\prime }=\left\langle \Psi _{n,m,\tilde{n},\tilde{m} }(\mathbf {R},\mathbf {r})\left| V(r)\right| \Psi _{n,m,\tilde{n}, \tilde{m}}(\mathbf {R},\mathbf {r})\right\rangle =\left\langle \tilde{\varphi } _{\tilde{n},\tilde{m}}^{(0)}(\mathbf {r})\left| V(r)\right| \tilde{ \varphi }_{\tilde{n},\tilde{m}}^{(0)}(\mathbf {r})\right\rangle , \end{aligned}$$

where $\tilde{\varphi }_{\tilde{n},\tilde{m}}^{(0)}(\mathbf {r})$ is given by Eq. (41). One can calculate the energies of a direct and an indirect PME using the Coulomb and Rytova-Keldysh potentials. By substituting these potentials into Eq. (19), and using the wavefunctions for the corresponding state, we obtain the analytical expressions for the eigenenergies $E_{0,0}^{\prime }$, $E_{0,1}^{\prime }$ and $E_{1,0}^{\prime }$. The total energy $\mathcal {E}_{n,\tilde{n},\tilde{m} }$ of a direct or indirect PME in strained and gapped graphene, taking into account $e-h$ attraction, is

$$\begin{aligned} \mathcal {E}_{n,\tilde{n},\tilde{m}}=\mathcal {E}_{0n,\tilde{n}}+E_{\tilde{n}, \tilde{m}}^{\prime }, \end{aligned}$$

where $\mathcal {E}_{0n,\tilde{n}}$ is given by Eq. (18), for the equal electron and hole masses in a mono or double layer strained and gaped graphene or by Eq. (15) for the double layer graphene with different masses of the electron and hole. Analytical expressions for the energy $E_{\tilde{n},\tilde{m}}^{\prime }$ of direct PMEs for the Coulomb and the RK potentials are given in SMs V B and V C. The energy of an indirect PME in a double layer of gapped graphene can be obtained by substituting the Coulomb or RK potentials into Eq. (19). In the case of the Coulomb potential the corresponding matrix elements can be evaluated analytically and the results are given in SM  V D. However, in the case of the Rytova-Keldysh potential the energy of an indirect PME in a double layer system could be found only numerically. The expressions for potentials that describe the interaction between the electron and hole which are located in different parallel graphene layers are given in SM V D1.

**Table 1:** Calculations performed for the monolayer strained and gapped graphene, $\Delta =0.25$ eV, and for a double layer strained graphene with the gap $\Delta =0.25$ eV in one layer and $\Delta =0.50$ eV in the other.

Energy	Potential	Monolayer	2 Layers	Landau level, meV	2 Layers	Landau level, meV
		$\Delta =0.25$ eV	$\Delta =0.25$ eV	$\Delta =0.25$ eV	$\Delta _{1}=0.25$ eV	$\Delta _{1}=0.25$ eV
					$\Delta _{2}=0.5$ eV	$\Delta _{2}=0.5$ eV
$E_{0,0}^{^{\prime }},$ meV	RK	27.001	21.185	131.3	28.402	98.4
	Coulomb	27.097	21.187		28.562	
$E_{0,1}^{^{\prime }},$ meV	RK	13.548	13.013	262.6	14.280	207.8
	Coulomb	13.549	13.014		14.281	
$E_{1,0}^{^{\prime }},$ meV	RK	20.228	15.128	262.6	21.264	185.9
	Coulomb	20.322	15.130		21.422	

The value of magnetic length *l* corresponds to $B/e=50$ T. Two strained and gapped graphene layers are separated by $D=1.7$ nm by the dielectric with $\varepsilon =13$.

In our calculations the uniform PMF *B*, acting on *e* or *h*, is related to the strain as $B=\frac{8\hbar \beta c}{a}$^1^, where $a=2.566\ {\mathring{A}}$ is the lattice constant^27^, and the strain parameters $\beta \approx 2$ and $c=10^{-3}$ m$^{-1}$ that are defined in Ref.^1^. It can be seen from Fig. 2 that the energies $E_{\tilde{n},\tilde{m}}^{^{\prime }}$ of PME’s are decreasing with the increase of the separation between graphene layers. Interesting enough, the comparison of results for the binding energies of the direct and indirect PME energies, calculated using the RK and Coulomb potential, for the parameters used are very close and almost the same. Also, the magnitudes of PME energies, calculated using the RK potential are a little bit smaller than ones, calculated using the Coulomb potential, because the RK potential implies the screening effects. The energies $E_{ \tilde{n},\tilde{m}}$ of indirect PMEs as a function of the separation *D* between gapped graphene layers are presented in Fig. 2. It can be seen, the energies of indirect PMEs decrease with the increase of *D* and increase with the increase of *B*. Note that dipolar excitons without magnetic field was analyzed in^28,29^ and their collective properties are analogous to dipolar ultracold atoms—see review^30 ^. ^31,32^ The results of calculations for the energies of the PMEs are given in Table 1. Following Ref.^19^ we perform calculations for the gap $\Delta _{1}=0.25$ eV and $\Delta _{2}=0.5$ eV. A gap in graphene is induced due to the breaking the sublattice symmetry. There are many ways to produce the gap. For example, by hydrogenation, which gives a gap of 1 eV^33^. Another proposal is to introduce a structured substrate, which can create a gap of 0.2–0.5 eV^19^ or a gap of 0.2 eV on a SiO$_{2}$ SiC sublattice^34^. Small band gaps have been observed, when graphene is grown on substrates SiC, i.e. 0.260 eV^5^ and 0.250 eV^6^ and gold on ruthenium (200 meV)^7^. The gap is indeed smaller on hBN substrates, typically 5–50 meV^35^. As it is mentioned before, the energies of the direct and indirect PMEs obtained using the RK and the Coulomb potentials are very close. The reason of this similarity is related to the fact that at large distances the RK potential has the asymptotic behavior of the Coulomb potential, while the screening effects, taken into account by Rytova-Keldysh potential, are quite weak. As a result the energy $E_{\tilde{n}, \tilde{m}}^{\prime }$ of a PME defined by (19) with functions $\tilde{\varphi }_{\tilde{n},\tilde{m}}^{(0)}(\mathbf {r})$ are comparable. However, there are significant differences for the energies of indirect PMEs obtained for a double layer system when both layers have the same gap $\Delta =0.25$ eV and when one layer has the gap $\Delta _{1}=0.25$ eV and the other one $\Delta _{2}=0.5$ eV. The difference for the Landau levels of indirect PMEs is due to the dependence of the effective PMF on $m_{e}$ and $m_{h}$.

From Eqs. (15) and (18) one can see that decrease of the gap leads to the increase of the energy levels. Therefore, the effect of a changing gap in the graphene spectrum on the pseudomagnetic excitons is that the effective exciton mass is an increasing function of the gap. Since the difference between the Landau levels is the cyclotron frequency, which is a decreasing function of the effective exciton mass, one concludes that the distance between the Landau levels is a decreasing function of the gaps. Moreover, our results for gaps $\Delta _{1}=0.25$ eV and $\Delta _{2}=0.5$ eV is a special case in which the the perturbation and the Schrödinger approaches are valid.

The analysis of the results presented in Table 1 show that even for the large gaps the perturbations by the Coulomb or RK potentials are small compared to the non-interacting eigenvalues. Therefore, our assumption that the Coulomb energy is a small perturbation compared to the non-interacting eigenvalues, is valid if the binding energy of a PME is much less than the distance between the Landau levels. At least two LL should exist inside these gaps which will be experimentally achievable^36^. In this case, mixing between different LLs can be neglected in evaluating Eq. (19).

## Strain-induced quantum Hall phenomena for PMEs

The QHE and especially the Fractional Quantum Hall effect (FQHE) has been discussed in length^37^. Given that the pseudomagnetic field acts like a magnetic field on the electrons, the standard theory of the QHE/FQHE can be applied without modification.

**Figure 3:**
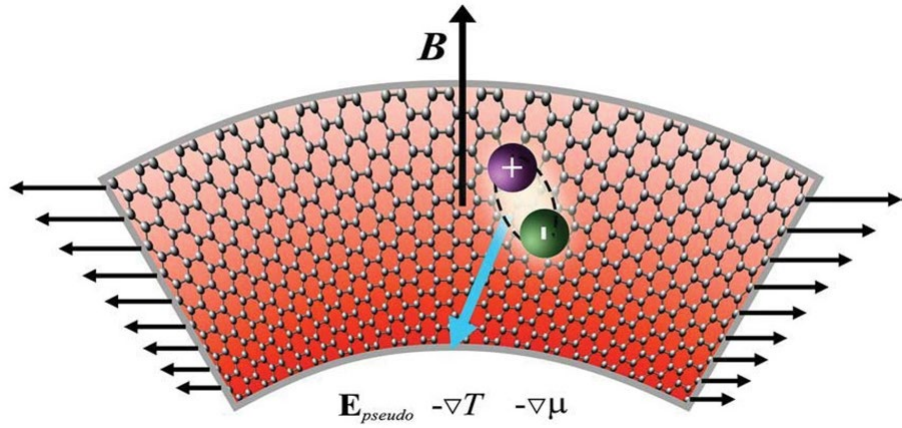
Conceptual picture of the quantum Hall effect of PMEs in graphene. The QHE PME flow, shown by the blue arrow, is generated by the PMF $\mathbf {B}$, normal to the graphene layer, and a pseudoelectric field $\mathbf {E}_{\mathrm {pseudo}}$ or $-\nabla T$, or $-\nabla \mu$, directed along the graphene layer. The QHE PME flow in the graphene layer is directed normal to $\mathbf {E}_{\mathrm {pseudo}}$ or $- \nabla T$, or $-\nabla \mu$. The strain is created by the normal forces applied at two opposite boundaries and their magnitude is gradually decreased, which is indicated by the length of the plotted arrows.

The conceptual picture of the quantum Hall effect of PMEs is presented in Fig. 3. The suggested bending geometry, when the graphene rectangle is bent into a circular arc, would generate a uniform PMF^2,38^. FQHE can be observed for PME in gauge field 15 Tesla as for 2D electrons in real magnetic field^39–41^. This field can be created by uniform stretching using polymer “muscles” as it was demonstrated recently^36^. While for some physical realizations the pseudomagnetic field caused by strain averages to zero (see e.g., Ref.^42^), we are focusing here on a uniform PMF on a finite but sufficiently large region. Let us estimate the conditions for the characteristic nonhomogeneous length or size of the system for observing the FQHE. Since the latter is equivalent to the IQHE for composite fermions, it is sufficient that the size of the system is substantially larger than the effective magnetic length *l* for composite fermions − by at least one order of magnitude. Therefore, it is sufficient for the characteristic energy of geometric quantization $\hbar ^{2}/MS,$ where *S* is the area of the system to be substantially less than the characteristic energies for composite fermions.

Another equivalent, more visual physical condition: the finiteness of the system is equivalent to the presence of an effective confining potential $kr^{2}/2=M\omega ^{2}r^{2}/2$. In the presence of a pseudomagnetic field, the problem becomes equivalent to the Fock-Darwin problem, but for an effective magnetic field acting on composite fermions. So to neglect the effect of the finite size it is sufficient to satisfy the inequality: $\omega _{c}\gg \omega$. It easy to show that both conditions are almost equivalent.

When graphene is under strain, shear strain induces a PMF, while the dilatation gives rise to an effective scalar potential which results in the pseudoelectric field, acting on the charge carriers independently of the sign of charge, contrary to ordinary electric field^2^. The pseudoelectric field $\mathbf {E}_{\mathrm { pseudo}}$ can be chosen to be normal to the PMF. The other option is to use laser illumination on the edge of samples, which creates gradients of the temperature *T* and/or chemical potential $\mu$. This drives electrons (*e* ) and holes (*h*) in the same direction. The latter can trigger the flow of PMEs without breaking the bound states of *e* and *h*. Below we show that $\mathbf {E}_{\mathrm {pseudo}}$ or $\nabla \mu$, or $\nabla T$ together with the PMF initiates the QHE for the PMEs. Note that, contrary to PMEs, the electric field normal to the magnetic field does not lead to any flow of magnetoexcitons, but only induces the dipole moments of magnetoexcitons. Lets us mention that $\nabla \mu$ and $\nabla T$ induce a flow of magnetoexcitons in the directions of the gradients, without creating Hall flows, in contrast to PMEs. A PMF leads to quantization of the PME spectrum which is discrete. Scattering effects due to random potentials and/or interaction between the PMEs would lead to a broadening of the LLs. The resulting multiband structure can be characterized by the Chern numbers of these PME bands, which can be observed in the transport properties. The strain-induced PMF causes a flow of the PMEs inside the graphene layers. The strain-induced pseudomagnetic and pseudoelectric field or existence of $\nabla \mu$, or $\nabla T$ give rise to the Hall effect for the PMEs, whose Hall conductivity is quantized according to the Chern numbers of the multiband structure. This can also be formulated in terms of an effective Ginzburg-Landau approach by coupling an additional statistical Chern-Simons gauge field to the bosonic PMEs^37,43^, where the resulting Hall conductivity is related to the Chern-Simons constant. Thus, analogously to the standard Integer Quantum Hall effect (IQHE) for the 2DEG in a magnetic field^26^, we obtain for the system of the PMEs in the presence of impurities the occurrence of the set of plateaus in the Hall resistivity $\varrho _{xy}$ and conductivity $\sigma _{xy}$ with quantized values: $\varrho _{xy} = -1/\sigma _{xy} = h/n$ ($n=1,2,3, \ldots$ is the LL for the motion of the center-of-mass of the PME). In the region of the plateau we have $\varrho _{xx} = \sigma _{xx} = 0$.

The degeneracy *d* of the LLs *n* is given by $d=2BS/\Phi _{0}$, where $\Phi _{0}=h/2$ is the quantum of pseudomagnetic flux and the factor 2 appears due to the same action of the PMF an electron and a hole. Thus, one can control the filling factor of the LL $\nu =N/d$ (*N* is the number of the PMEs) either by changing the strain, inducing the PMF, or by laser pumping changing the number *N* of the PMEs. So one can observe not only the IQHE but also the FQHE for PMEs. For example, to observe the FQHE at the filling factor $\nu =1/3$, it occurs that one needs the PMF *B* corresponding to four (but not three as for the 2DEG in a magnetic field) quanta of pseudomagnetic flux accounting for one bosonic PME. In this case, a composite fermion can be formed via attachment of one pseudomagnetic flux quantum to one PME, and these composite fermions with three remaining pseudomagnetic flux quanta form the FQHE state at $\nu =1/3$ analogously to the FQHE for the 2DEG in a magnetic field^44^. Note that we also can achieve the state of the PME system analogous to the state of composite fermions at the filling factor $\nu =1/2$^45^, when the filling factor of PMEs $\nu =1/3$. Really the latter corresponds to three pseudomagnetic flux quanta on one PME. When one pseudomagnetic flux quantum is attached to each boson, PME, one obtains the system of the composite fermions at the filling factor $\nu =1/2$ (experimentally observable composite fermions, analogously to electrons with two attached magnetic flux quanta^45^, correspond to the PMEs with three attached pseudomagnetic flux quanta). Thus, for the neutral PMEs (bosons) one can observe the IQHE, FQHE, and $\nu =1/2$ phenomena similar to the ones for charged electrons, forming 2DEG in the high magnetic field^44^.

The characteristic time necessary for the formation of the FQHE state can be estimated as $t\sim \hbar /\Delta E$, where $\Delta E$ is characteristic energy defined the stability of FQHE (see below) - the energy difference between the state with one hole excitation and the ground state of the system (described by the Laughlin-type wave function). The estimate of $\Delta E$ below shows that this time is essentially smaller than the lifetime of the exciton. This allows the observation of FQHE for PMEs. The allowed temperatures for the observation of FQHE are $T<\Delta E/k_{B}$. Note that there are different estimates of the gap $\Delta E$ associated with different excitations over the ground state of the system in the FQHE state- i. the creation of a composite hole; ii. the creation of a Coulomb interacting e-h pair; iii. the creation of a collective excitation of the pseudoskyrmions type. The estimates for different excitations do not coincide, but they are of the same order of magnitude! (see Ref.^46^ and references therein).

## Discussion and conclusions

**Figure 4:**
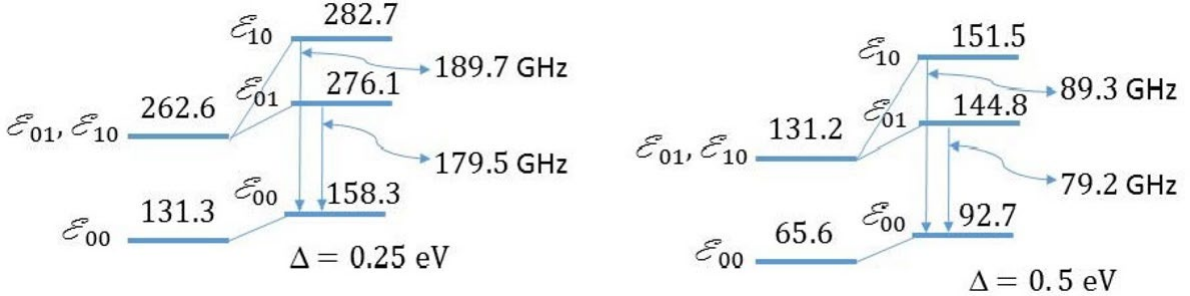
Schematic diagrams for the energy levels and transitions of the direct PME in the strained monolayer graphene. Calculations performed using RK potential for the value of magnetic length *l* that corresponds to $B/e=50\ \mathrm {T}$ and for $\Delta =0.25$ eV and $\Delta =0.5$ eV. The energy levels are given in meV. The diagrams are not drawn to scale.

Let us now estimate the characteristic energy of interaction of excitons at distances determined by the filling of the Landau level. For the FQHE these are distances of the order of several pseudomagnetic lengths *l*. The interaction energy (and consequently the stability of FQHE states) is greater for spatially indirect excitons. Spatially indirect excitons (with electron and hole in different layers) have crossover from a Coulomb interaction (on distances of the order of PME radius $a=l$) to a weaker dipole-dipole interaction (on distances much larger than PME radius *l*). So on the distances of the order of PME radius *l* one can use for the estimation of the indirect excitons interaction and the stability of corresponding composite fermion state the same expressions as for electrons, i.e. the energy difference between the state with one composite hole excitation and the ground state of the system (described by the Laughlin wave function) for $\nu = 1/3$ is $\Delta E \sim 0.02/(\varepsilon _{d} l)$, where $\varepsilon _{d}$ is the dielectric susceptibility of the surrounding media (see Ref.^46^ and references therein); see also Ref.^47^ and references therein); this estimate is in agreement with experimental data for $\nu = 1/3$ (see Ref.^48^ and references therein).

So for initial bosons (PMEs) with Landau level filling factor $\nu =1/4$ and resulting composite fermion (PMEs with attached pseudomagnetic field flux quantum) with Landau level filling factor $\nu =1/3$, we have $\Delta E \sim 0.02/(4 \varepsilon _{d} l)$.

The stability of the integer quantum Hall effect for PMEs is determined by the energy gap between Landau levels, which is proportional to the pseudomagnetic field. Landau level quantization for PMEs can be revealed by optical spectroscopy. To reveal IQHE Hall quantization by transport experiments for PMEs the following are needed: i. the plateau formation due to localization on impurities; ii. all states at Landau level must be filled. The latter is impossible for Bose quasiparticles, but for PMEs it is possible only for composite fermions formed due to exciton-exciton interactions. The composite fermion stability is also defined by the energy gap $\Delta E$ proportional to the characteristic energy of interaction of excitons $\Delta E$ at a distance $r=l$ corresponding to the filling of the Landau level $\nu =1$. Thus the energy corresponding to forming IQHE plateau in contrast to fermion, electron system has the order of $\Delta E\sim e^{2}/\varepsilon _{d}l$ and the necessary temperatures for the observation IQHE for PMEs in transport experiments are $T < \Delta E/k_{B}$.

In conclusion, we propose the zero magnetic field quantum Hall phenomena for excitons in a high PMF. The Hall valley flows of direct and indirect PME’s, similar to Hall currents of charged particles, can be excited in a mono or double layer of the gapped graphene, respectively. In order to observe the quantum Hall effect for PMEs, one has to measure the PME flows. We suggested the existence of PMEs and calculated their properties. Moreover, we considered the variety of collective properties of PMEs analogous to 2DEG: IQHE, FQHE, and the state of composite fermions at $\nu =1/2$. The spectrum of direct and indirect PMEs can be studied by using the photoluminescence.

The observation of LLs quantization and the gap $\Delta E$ in FQHE state for PMEs can be achieved by two ways: i. using a THz spectroscopy for transitions between neighboring LLs in the same band; ii. using an optical spectroscopy for transitions between LLs of neighboring bands with $\left| n_{1}-n_{2}\right| =1,$ where $n_{1}$ and $n_{2}$ are quantum numbers of LLs in lower and upper band, respectively. Fractional statistics of composite fermions can be revealed in interference experiments. There are two possible spectroscopical manifestations of Landau quantization and PME formation for intraband transition between filled and empty pseudo Landau levels in THz spectral region and interband transition between filled and empty pseudo Landau levels in IR spectral region. The difference is due to the interband energy gap denoted as the gap $\Delta$ estimated for calculations as 0.25 eV or 0.5 eV. In the absorption spectrum one can observe the resonance, caused by the transitions between the Landau levels. Initially, the resonance in the emission spectrum is caused by the recombination of the free electron-hole pairs. Subsequently after the PME formation the exciton recombination contributes to the photoluminescence spectrum. These possible resonances in the radiation spectrum due to the transitions between PME energy levels are represented in Fig. 4. According to Fig. 4, the emission spectrum of the direct PMEs in a single graphene layer depends on the gap. The emission frequencies are the order of 190–180 GHz for $\Delta = 0.25$ eV and 90–80 GHz for $\Delta = 0.5$ eV.

The experimental observation of FQHE states for PMEs is possible at temperatures $T<\Delta E/k_{B}$, where $k_{B}$ is the Boltzmann constant and $\Delta E$ is the energy between the excited and ground states of the system. For the observation of FQHE for indirect excitons in suspended double layer graphene in PMF $B/e\sim 20\ \mathrm {T}$ at the lowest FQHE plateau (at Landau filling $\nu =1/k$ with lowest *k* and small interexciton separations), where the interaction between indirect excitons are almost the Coulomb one, plausible temperatures are the same as for the FQHE for electrons, i.e. order of $20\ \mathrm {K.}$ This is the most optimal value for experiments. The plausible temperature for the double layer in the surrounding media with dielectric susceptibility $\varepsilon _{d}$ is order of $20\ \mathrm {K}/\varepsilon _{d}$ (see Ref.^48^ and references therein). It is worth to notice that for the larger *k* indirect exciton interaction is weaker and corresponding $\Delta E$ and temperatures are lower and for direct PMEs even more lower due to weaker exciton-exciton interactions.

The intervalley excitons give a hierarchy of states of the integer and fractional quantum Hall effect, as disscused above. Thus, after the formation of excitons, two subsystems with radically different properties can arise. Their mutual influence will not be considered in this article. Bright excitons will quickly die out and only a system of dark excitons survive. It is possible to increase the lifetime of bright excitons using the engineering of the optical cavity, as suggested in Ref.^49^. Experimental observation of dark excitons can be done by using Förster resonant energy transfer from a dark exciton to neighboring light excitons or molecules (for example, adsorbed on graphene, or in the van der Waals structure of a graphene—2D molecular crystal), etc.^50^.

The results of our study could provide a novel route to quantum Hall physics of PMEs valleytronics in graphene. The presented Quantum Hall phenomena for the PMEs are important, since they imply that the Quantum Hall physics can be observed in the novel system of neutral bosons without magnetic field. Besides, the system considered due to its discrete spectra can be used as a new realization for a qubit system for quantum technologies.

## Supplementary Information

Supplementary Information.
