# Supplementary material for: Strain-induced quantum Hall phenomena of excitons in graphene
Source: Sci Rep. 2022 Feb 22;12:2950. doi: 10.1038/s41598-022-06486-z (PMC8863812; doi:10.1038/s41598-022-06486-z)
Supplement: Supplementary file 1 — Supplementary Information. [file 41598_2022_6486_MOESM1_ESM.pdf]

Strain-induced quantum Hall phenomena of excitons in graphene

Oleg L. Berman[1,2], Roman Ya. Kezerashvili[1,2,*], Yurii E. Lozovik[3,4], and Klaus G. Ziegler[5]
[1] *Physics Department, New York City College of Technology, The City University of New York, Brooklyn, NY 11201, USA*
[2] *The Graduate School and University Center, The City University of New York, New York, NY 10016, USA*
[3] *Institute of Spectroscopy, Russian Academy of Sciences, 142190 Troitsk, Moscow, Russia*
[4] *Research University Higher School of Economics, Moscow, Russia 101000*
[5] *Institut für Physik, Universität Augsburg D-86135 Augsburg, Germany*
[*] *Corresponding author Email:rkezerashvili@citytech.cuny.edu*

I. SUPPLEMENTARY MATERIAL

A. Eigenvalues and eigenfunctions

In expanded form the operator $\hat{\mathbf{P}}^2$ is given by

$$\hat{\mathbf{P}}^2 = -\hbar^2\nabla^2_{\mathbf{R}} + 2i\hbar\left(\mathbf{B}\times\mathbf{R}\right)\cdot\nabla_{\mathbf{R}} + B^2R^2 + i\hbar\gamma\left(\mathbf{B}\times\mathbf{r}\right)\cdot\nabla_{\mathbf{R}} + \gamma\left(\mathbf{B}\times\mathbf{R}\right)\cdot\left(\mathbf{B}\times\mathbf{r}\right) + \frac{\gamma^2B^2r^2}{4}. \tag{1}$$

In the center-of-mass reference system the Hamiltonian (6) from the main text can be written in the following form:

$$\hat{\mathcal{H}}_0 = \hat{D}_1 + \hat{D}_2 + \hat{D}_3, \tag{2}$$

where

$$\hat{D}_1 = -\frac{\hbar^2\nabla^2_{\mathbf{R}}}{2M} - \frac{\hbar^2\nabla^2_{\mathbf{r}}}{2\mu}, \tag{3}$$

$$\hat{D}_2 = \frac{i\hbar}{2}\left[\frac{2\left(\mathbf{B}\times\mathbf{R}\right)\cdot\nabla_{\mathbf{R}}}{M} + \frac{\left(m_h-m_e\right)\left(\mathbf{B}\times\mathbf{R}\right)\cdot\nabla_{\mathbf{r}}}{m_em_h} + \frac{\gamma\left(\mathbf{B}\times\mathbf{r}\right)\cdot\nabla_{\mathbf{R}}}{M} + \frac{\left(m_e^2+m_h^2\right)\left(\mathbf{B}\times\mathbf{r}\right)\cdot\nabla_{\mathbf{r}}}{m_em_hM}\right], \tag{4}$$

$$\hat{D}_3 = \frac{B^2}{8}\left[\frac{R^2}{\mu} + \frac{2(m_h-m_e)\left(\mathbf{r}\cdot\mathbf{R}\right)}{m_em_h} + \frac{\left(m_e^3+m_h^3\right)r^2}{m_em_h(m_e+m_h)^2}\right]. \tag{5}$$

and M and μ are the total and reduced exciton masses, respectively, given by

$$M = m_e + m_h, \qquad \frac{1}{\mu} = \frac{1}{m_e} + \frac{1}{m_h}. \tag{6}$$

Eqs. (2)-(6) are used to derive $\hat{\mathcal{H}}_0$ in the form of Eq. (12) from the main text.

Let us find the eigenfunctions of the operator $\hat{\tilde{\mathcal{H}}}_0$ defined as

$$\hat{\tilde{\mathcal{H}}}_0 = \frac{\hat{\mathbf{P}}^2}{2M} = \frac{1}{2M}\left[-i\hbar\nabla_{\mathbf{R}} - \mathbf{B}\times\mathbf{R} - \mathbf{A}_0\right]^2, \tag{7}$$

where the vector $\mathbf{A}_0$ is given by

$$\mathbf{A}_0 = \frac{\gamma\mathbf{B}\times\mathbf{r}}{2}. \tag{8}$$

The eigenfunctions and eigenvalues of the Hamiltonian are considered for the both cases $\mathbf{A}_0 = 0$ and $\mathbf{A}_0 \neq 0$. If $\mathbf{A}_0 = 0$, the eigenfunction of $\widetilde{H}_0$ is given by $\psi^{(0)} = \psi^{(0)}_{n,m}(\mathbf{R})$, which is the wavefunction for a free particle of unit charge in the effective PMF $2\mathbf{B}$ in the cylindrical gauge in eigenvalue E_n of $\hat{\mathbf{P}}^2$ is defined as [5]

$$E_n = \frac{P_n^2}{2M} = \left(n+\frac{1}{2}\right)\hbar\omega_c, \tag{9}$$

where $\omega_c = 2B/M$ is the cyclotron frequency for the motion of the center-of-mass of a non-interacting electron-hole pair. The quantum numbers $n = 0, 1, 2, \ldots$ and $m = 0, 1, 2, \ldots$ for $\psi^{(0)} = \psi^{(0)}_{n,m}(\mathbf{R})$ and in Eq. (9) are related to the motion of the center-of-mass of a non-interacting electron and hole.

If $\mathbf{A}_0 \neq 0$, we define the scalar function $f(\mathbf{R}, \mathbf{r})$ so that $\mathbf{A}_0 \equiv \nabla_{\mathbf{R}} f(\mathbf{R}, \mathbf{r})$ and we have $f(\mathbf{R}, \mathbf{r}) = \mathbf{A}_0 \cdot \mathbf{R}$. The Schrödinger equation is invariant with respect to the translation of the coordinate $\mathbf{R}$ and simultaneous gauge transformation. In this case the eigenvalue of $\hat{\mathbf{P}}^2$ is the same as the eigenvalue at $\mathbf{A}_0 = 0$ given by $E_n = E_n^{(0)} = P_n^2/(2M)$, and the eigenfunction of $\hat{\mathbf{P}}^2$ denoted as ψ is given by

$$\psi \equiv \psi_{n,m}(\mathbf{R}, \mathbf{r}) = \psi^{(0)}_{n,m}(\mathbf{R}) e^{if(\mathbf{R},\mathbf{r})/\hbar}. \tag{10}$$

We can see that

$$e^{if(\mathbf{R},\mathbf{r})/\hbar} = e^{i\mathbf{A}_0\cdot\mathbf{R}/\hbar} = e^{i\gamma(\mathbf{B}\times\mathbf{r})\cdot\mathbf{R}/2\hbar} = e^{i\gamma(\mathbf{B}\times\mathbf{R})\cdot\mathbf{r}/2\hbar}. \tag{11}$$

By substituting Eq. (11) into Eq. (10), one obtains

$$\psi \equiv \psi_{n,m}(\mathbf{R}, \mathbf{r}) = \psi^{(0)}_{n,m}(\mathbf{R}) e^{i\gamma(\mathbf{B}\times\mathbf{r})\cdot\mathbf{R}/2\hbar}. \tag{12}$$

If $\mathbf{A}_0 \neq 0$, by substituting ψ from Eq. (12) and $\hat{\mathbf{P}}^2$ from Eq. (14) from the main text into $\hat{\tilde{\mathcal{H}}}_0\psi = E^{(0)}\psi$, one obtains

$$\frac{1}{2M}\left[-i\hbar\nabla_{\mathbf{R}} - \mathbf{B}\times\mathbf{R}\right]^2 \psi^{(0)}_{n,m}(\mathbf{R}) = E_n^{(0)}\psi^{(0)}_{n,m}(\mathbf{R}). \tag{13}$$

The eigenfunction ψ of the Hamiltonian $\mathcal{H}_0$ is given by

$$\psi = \psi^{(0)}_{n,m}(\mathbf{R}) e^{i\gamma(\mathbf{B}\times\mathbf{R})\cdot\mathbf{r}/2\hbar}\Phi(\mathbf{r}). \tag{14}$$

The function $\Phi(\mathbf{r})$ can be obtained from the solution of the following equation:

$$\left[E_n - \frac{1}{2Mm_em_h}\left(\hbar M\nabla_{\mathbf{r}} - i\mathbf{S}(\mathbf{R}, \mathbf{r})\right)^2\right] e^{i\gamma(\mathbf{B}\times\mathbf{R})\cdot\mathbf{r}/2\hbar}\Phi(\mathbf{r}) = \mathcal{E}_0 e^{i\gamma(\mathbf{B}\times\mathbf{R})\cdot\mathbf{r}/2\hbar}\Phi(\mathbf{r}). \tag{15}$$

$\mathcal{E}_0 = E_n + \tilde{E}$ is the eigenvalue of the Hamiltonian $\hat{\mathcal{H}}_0$ (12) from the main text. $\tilde{E}$ and $\Phi(\mathbf{r})$ can be obtained from the solution of the following equation:

$$\left[-\frac{1}{2Mm_em_h}\left(\hbar M\nabla_{\mathbf{r}} - i\mathbf{S}(\mathbf{R}, \mathbf{r})\right)^2\right] e^{i\gamma(\mathbf{B}\times\mathbf{R})\cdot\mathbf{r}/2\hbar}\Phi(\mathbf{r}) = \tilde{E} e^{i\gamma(\mathbf{B}\times\mathbf{R})\cdot\mathbf{r}/2\hbar}\Phi(\mathbf{r}). \tag{16}$$

Equation (16) can be rewritten as

$$\frac{1}{2\mu}\left(-i\hbar\nabla_{\mathbf{r}} - \frac{\left(m_e^2 + m_h^2\right)\mathbf{B}\times\mathbf{r}}{2M^2} - \frac{\gamma\mathbf{B}\times\mathbf{R}}{2}\right)^2 \tilde{\Phi}(\mathbf{R}, \mathbf{r}) = \tilde{E}\tilde{\Phi}(\mathbf{R}, \mathbf{r}). \tag{17}$$

Equation (17) is invariant with respect to the translation of the coordinate $\mathbf{r}$ and simultaneous gauge transformation. In this case, $\tilde{\Phi}(\mathbf{R}, \mathbf{r})$ is defined as

$$\tilde{\Phi}(\mathbf{R}, \mathbf{r}) \equiv e^{i\gamma(\mathbf{B}\times\mathbf{R})\cdot\mathbf{r}/2\hbar}\Phi(\mathbf{r}). \tag{18}$$

By substituting $\tilde{\Phi}(\mathbf{R}, \mathbf{r})$ from Eq. (18) into Eq. (17), one obtains

$$\frac{1}{2\mu}\left(-i\hbar\nabla_{\mathbf{r}} - \frac{\left(m_e^2 + m_h^2\right)\mathbf{B}\times\mathbf{r}}{2M^2}\right)^2 \Phi(\mathbf{r}) = \tilde{E}\Phi(\mathbf{r}). \tag{19}$$

Equation (19) presents the equation for the harmonic oscillator with the cyclotron frequency $\tilde{\omega}_c = \left(m_e^2 + m_h^2\right)\mathbf{B}/\left(M^2\mu\right)$ for the relative motion of a non-interacting electron-hole pair. The corresponding eigenvalues read

$$\tilde{E}_{\tilde{n}} = \left(\tilde{n} + \frac{1}{2}\right)\hbar\tilde{\omega}_c, \tag{20}$$

and for eigenfunctuions of the operator $\hat{\mathcal{H}}_0$ given by Eq. (12) from the main text, we get

$$\tilde{\Phi}(\mathbf{R},\mathbf{r}) = \tilde{\varphi}^{(0)}(\mathbf{r}) e^{i\gamma(\mathbf{B}\times\mathbf{r})\cdot\mathbf{R}/2\hbar}. \tag{21}$$

where $\tilde{\varphi}^{(0)}_{\tilde{n},\tilde{m}}(\mathbf{r})$ is the wavefunction for a free particle of unit charge the effective PMF $\tilde{\mathbf{B}} = \left(m_e^2 + m_h^2\right)\mathbf{B}/M^2$ in the cylindrical gauge [1, 2, 5]:

$$\tilde{\varphi}^{(0)}_{\tilde{n},\tilde{m}}(\mathbf{r}) = \left[\frac{\tilde{n}!}{2\pi\left(\tilde{n}+|\tilde{m}|\right)!}\right]^{1/2} \frac{\exp\left(i\tilde{m}\phi\right)}{l} \left(\frac{r}{\sqrt{2}l}\right)^{|\tilde{m}|} L_{\tilde{n}}^{|\tilde{m}|}\left(\frac{r^2}{2l^2}\right) \exp\left(-\frac{r^2}{4l^2}\right) \ , \tag{22}$$

where $l = \sqrt{\hbar/\tilde{B}}$ is the pseudomagnetic length. In Eq. (22), $L_{\tilde{n}}^{|\tilde{m}|}$ denotes Laguerre polynomials. Let us mention that l is measured in m, since $\tilde{B}$ is measured in kg/s. Note that we consider a PME formed by an electron and a hole located in the same type of valley, e.g., in the point K (or K') of the Brillouin zone.

Combining Eqs. (14), (18), and (21), one can see that the wavefunction of the electron-hole pair in the strain-induced PMF, neglecting the electron-hole attraction, can be written as

$$\Psi_{n,m,\tilde{n},\tilde{m}}(\mathbf{R},\mathbf{r}) = \psi^{(0)}_{n,m}(\mathbf{R})\tilde{\varphi}^{(0)}_{\tilde{n},\tilde{m}}(\mathbf{r}) e^{i\gamma(\mathbf{B}\times\mathbf{r})\cdot\mathbf{R}/2\hbar}, \tag{23}$$

where γ is defined by Eq. (11) from the main text, $\psi^{(0)}_{n,m}(\mathbf{R})$ is the wavefunction for a free particle in the effective PMF $2\mathbf{B}$ in the cylindrical gauge [1, 2, 5], $\tilde{\varphi}^{(0)}_{\tilde{n},\tilde{m}}(\mathbf{r})$ is the wavefunction for a free particle the effective pPMF $\tilde{\mathbf{B}} = \left(m_e^2 + m_h^2\right)\mathbf{B}/M^2$ in the cylindrical gauge.

B. The energy of a PME

To find the energy of a direct and indirect exciton PME one should evaluate the following matrix elements

$$E_{0,0} = 2\pi \int_0^{+\infty} \left[\tilde{\varphi}^{(0)}_{0,0}(\mathbf{r})\right]^2 V(r) r dr, \tag{24}$$

$$E_{0,1} = \int_0^{2\pi} d\phi \int_0^{+\infty} r dr \left[\tilde{\varphi}^{(0)}_{0,1}(\mathbf{r})\right]^2 V(r), \tag{25}$$

$$E_{1,0} = 2\pi \int_0^{+\infty} \left[\tilde{\varphi}^{(0)}_{1,0}(\mathbf{r})\right]^2 V(r) r dr, \tag{26}$$

where

$$\tilde{\varphi}^{(0)}_{0,0}(\mathbf{r}) = \left[\frac{1}{2\pi}\right]^{1/2} \frac{1}{l} \exp\left(-\frac{r^2}{4l^2}\right), \tag{27}$$

$$\tilde{\varphi}^{(0)}_{0,1}(\mathbf{r}) = \left[\frac{1}{2\pi}\right]^{1/2} \frac{\exp\left(i\phi\right)}{l} \left(\frac{r}{\sqrt{2}l}\right) \exp\left(-\frac{r^2}{4l^2}\right), \tag{28}$$

$$\tilde{\varphi}^{(0)}_{1,0}(\mathbf{r}) = \left[\frac{1}{2\pi}\right]^{1/2} \frac{1}{l} \left(1 - \frac{r^2}{2l^2}\right) \exp\left(-\frac{r^2}{4l^2}\right) \tag{29}$$

and $V(r)$ is the Coulomb or RK potential. In the case of an indirect PME in the potential $V(r)$ the corresponding interparticle distance should be replaced by the expression $\sqrt{r^2+D^2}$ [6–9], where D is the interlayer separation.

C. The energy for direct PME's for the Coulomb and Rytova-Keldysh potentials

The energy of a direct PME in a monolayer of gapped graphene double layer can be calculated by substituting the Coulomb potential into Eq. (6) and one obtains

$$E_{0,0} = -E_0; \quad E_{0,1} = -\frac{E_0}{2}; \quad E_{1,0} = -\frac{3E_0}{4}. \tag{30}$$

In Eq. (30) E_0 is given by

$$E_0 = \frac{ke^2}{\varepsilon_d l}\sqrt{\frac{\pi}{2}}, \tag{31}$$

where $l = \sqrt{\hbar/\tilde{B}}$ is the pseudomagnetic length.

The analytical expressions for the energy of a direct PME obtained using the Rytova-Keldysh (RK) potential [3, 4] are the following:

$$E_{0,0} = \frac{\pi k e^2}{(\varepsilon_1+\varepsilon_2)\,\rho_0}\left[e^{-\frac{l^2}{2\rho_0^2}}\mathrm{Erfi}\left(\frac{l}{\sqrt{2}\rho_0}\right) - G\left(\left\{\{0\},\{-\frac{1}{2}\}\right\},\left\{\{0,0\},\{-\frac{1}{2}\}\right\};\frac{l^2}{2\rho_0^2}\right)\right]. \tag{32}$$

$$E_{0,1} = \frac{\pi k e^2}{(\varepsilon_1+\varepsilon_2)\,\rho_0}\frac{1}{2\pi\rho_0^2}e^{-\frac{l^2}{2\rho_0^2}}\left[-e^{\frac{l^2}{2\rho_0^2}}(2\rho_0^2-\sqrt{2\pi}\rho l) - \pi(l^2-2\rho^2)\mathrm{Erfi}\left(\frac{l}{\sqrt{2}\rho_0}\right) + (l^2-2\rho_0^2)\mathrm{Ei}\left(\frac{l^2}{2\rho_0^2}\right)\right] \tag{33}$$

$$E_{1,0} = E_{0,0} - \frac{\pi k e^2}{(\varepsilon_1+\varepsilon_2)\,\rho_0}\frac{1}{4\pi\rho_0^4}e^{-\frac{l^2}{2\rho_0^2}}\left[l^4(\gamma-1) + l^3\rho\sqrt{2\pi}e^{\frac{l^2}{2\rho_0^2}} - 2l^2\rho^2(1+\gamma) - e^{\frac{l^2}{2\rho_0^2}}(7\sqrt{2\pi}l\rho^3 - 12\rho^4)\right. \tag{34}$$

$$-(6l^2\rho^2-8\rho^4)\mathrm{Ei}\left(\frac{l^2}{2\rho_0^2}\right) - \pi(l^4-8l^2\rho^2+8\rho^4)\mathrm{Erfi}\left(\frac{l}{\sqrt{2}\rho_0}\right) - (l^4+2l^2\rho^2)\ln 2 + 2(l^4-2l^2\rho^2)\ln\frac{l}{\rho} \tag{35}$$

$$\left.+e^{\frac{l^2}{2\rho_0^2}}\left(\sqrt{2}l\rho_0 - \rho_0^2 l^2\,{}_1F_1\left(2,1;\frac{l^2}{2\rho_0^2}\right)\right)\right], \tag{36}$$

where γ is Euler constant, Erfi(x) is the imaginary error function, the Maijer $G-$function, Ei(x) is the exponential integral function, and ${}_1F_1\,(a,b;x)$ is the Kummer confluent hypergeometric function.

D. The energy for indirect PME's for the Coulomb potential

$$E_{0,0}(D) = -E_0 \exp\left[\frac{D^2}{2l^2}\right]\mathrm{Erfc}\left[\frac{D}{\sqrt{2}l}\right], \tag{37}$$

$$E_{0,1}(D) = -E_0\left[\left(\frac{1}{2}-\frac{D^2}{2l^2}\right)\exp\left[\frac{D^2}{2l^2}\right]\mathrm{Erfc}\left[\frac{D}{\sqrt{2}l}\right] + \frac{D}{\sqrt{2\pi}l}\right], \tag{38}$$

$$E_{1,0}(D) = -E_0\left[\left(\frac{3}{4}+\frac{D^2}{2l^2}+\frac{D^4}{4l^4}\right)\exp\left[\frac{D^2}{2l^2}\right]\mathrm{Erfc}\left[\frac{D}{\sqrt{2}l}\right] - \frac{D}{2\sqrt{2\pi}l} - \left(\frac{D}{\sqrt{2}l}\right)^3\frac{1}{\sqrt{\pi}}\right], \tag{39}$$

where Erfc(x) is the complementary error function and E_0 is given by (31). These expressions partially concise with the expressions obtained in the case of uniform magnetic field [2].

1. *Interactions in double layer*

The potential energy of $e-h$ attraction $V\left(|\mathbf{r}_e-\mathbf{r}_h|\right)$ in Eq. (5) from the main text can be described by the Rytova-Keldysh [3, 4] or Coulomb potentials. The corresponding expressions that describe the interaction between the electron and hole which are located in different parallel graphene monolayers are the following:

$$V_{RK}(\sqrt{r^2+D^2}) = -\frac{\pi k e^2}{2\kappa\rho_0}\left[H_0\left(\frac{\sqrt{r^2+D^2}}{\rho_0}\right) - Y_0\left(\frac{\sqrt{r^2+D^2}}{\rho_0}\right)\right], \tag{40}$$

for the RK potential [8, 9], and

$$V_C\left(\sqrt{r^2+D^2}\right) = -\frac{ke^2}{\kappa\left(\sqrt{r^2+D^2}\right)} \tag{41}$$

for the Coulomb potential. In Eqs. eq:indkeld and eq:indcoul D is the separation between two graphene layers. The results of our calculations are compared with the calculation, employing the Coulomb potential.

[1] Lerner, I. V. & Lozovik, Yu. E. Mott exciton in a quasi-two-dimensional semiconductor in a strong magnetic field, *Sov. Phys. JETP* **51**, 588 (1980).

[2] Lozovik, Yu. E. & Ruvinsky, A. M. Magnetoexcitons in coupled quantum wells, *Phys. Lett. A* **227**, 271 (1997). https://doi.org/10.1016/S0375-9601(97)00039-X

[3] Rytova, N. S. The screened potential of a point charge in a thin film, *Proc. Moscow State University, Phys. Astron.* **3**, 30 (1967).

[4] Keldysh, L. V. Coulomb interaction in thin semiconductor and semimetal films, *JETP Lett.* **29**, 658 (1979).

[5] Landau, L. D. & Lifshitz, E. M. *Quantum Mechanics: Non-Relativistic Theory* (Pergamon, Oxford, 1977).

[6] Berman O. L. & Kezerashvili, R. Ya. Superfluidity of dipolar excitons in a transition metal dichalcogenide double layer, *Phys. Rev. B* **96**, 094502 (2017). https://doi.org/10.1103/PhysRevB.96.094502

[7] Berman, O. L., Gumbs, G., & Kezerashvili, R. Ya. Bose-Einstein condensation and superfluidity of dipolar excitons in a phosphorene double layer, *Phys. Rev. B* **96**, 014505 (2017). https://doi.org/10.1103/PhysRevB.96.014505

[8] Kezerashvili, R. Ya. & Spiridonova, A. Effects of parallel electric and magnetic fields on rydberg excitons in buckled two-dimensional materials, *Phys. Rev. B* **103**, 165410 (2021). doi: 10.1103/PhysRevB.103.165410

[9] Kezerashvili, R. Ya. & Spiridonova, A. Magnetoexcitons in transition metal dichalcogenides monolayers, bilayers, and van der waals heterostructures, *Phys. Rev. Research* **3**, 033078 (2021). doi: 10.1103/PhysRevResearch.3.033078
